# Abuse risks and routes of administration of different prescription opioid compounds and formulations

**DOI:** 10.1186/1477-7517-8-29

**Published:** 2011-10-19

**Authors:** Stephen F Butler, Ryan A Black, Theresa A Cassidy, Taryn M Dailey, Simon H Budman

**Affiliations:** 1 Inflexxion, Inc. 320 Needham St. Suite 100, Newton, MA 02464, USA

## Abstract

**Background:**

Evaluation of tamper resistant formulations (TRFs) and classwide Risk Evaluation and Mitigation Strategies (REMS) for prescription opioid analgesics will require baseline descriptions of abuse patterns of existing opioid analgesics, including the relative risk of abuse of existing prescription opioids and characteristic patterns of abuse by alternate routes of administration (ROAs). This article presents, for one population at high risk for abuse of prescription opioids, the unadjusted relative risk of abuse of hydrocodone, immediate release (IR) and extended release (ER) oxycodone, methadone, IR and ER morphine, hydromorphone, IR and ER fentanyl, IR and ER oxymorphone. How relative risks change when adjusted for prescription volume of the products was examined along with patterns of abuse via ROAs for the products.

**Methods:**

Using data on prescription opioid abuse and ROAs used from 2009 Addiction Severity Index-Multimedia Version (ASI-MV^®^) Connect assessments of 59,792 patients entering treatment for substance use disorders at 464 treatment facilities in 34 states and prescription volume data from SDI Health LLC, unadjusted and adjusted risk for abuse were estimated using log-binomial regression models. A random effects binary logistic regression model estimated the predicted probabilities of abusing a product by one of five ROAs, intended ROA (i.e., swallowing whole), snorting, injection, chewing, and other.

**Results:**

Unadjusted relative risk of abuse for the 11 compound/formulations determined hydrocodone and IR oxycodone to be most highly abused while IR oxymorphone and IR fentanyl were least often abused. Adjusting for prescription volume suggested hydrocodone and IR oxycodone were least often abused on a prescription-by-prescription basis. Methadone and morphine, especially IR morphine, showed increases in relative risk of abuse. Examination of the data without methadone revealed ER oxycodone as the drug with greatest risk after adjusting for prescription volume. Specific ROA patterns were identified for the compounds/formulations, with morphine and hydromorphone most likely to be injected.

**Conclusions:**

Unadjusted risks observed here were consistent with rankings of prescription opioid abuse obtained by others using different populations/methods. Adjusted risk estimates suggest that some, less widely prescribed analgesics are more often abused than prescription volume would predict. The compounds/formulations investigated evidenced unique ROA patterns. Baseline abuse patterns will be important for future evaluations of TRFs and REMS.

## Background

This article uses self-report data collected from individuals entering substance abuse treatment from a large number of treatment facilities across the country to examine the relative risks of abuse of specific prescription opioid compounds and formulations and to describe route of administration (ROAs) patterns that are characteristic of the different opioid compounds and formulations. A more comprehensive understanding of the abuse patterns of these medications is critical to inform current public health efforts intended to manage the risk for abuse of these important medications. While long-term opioid therapy for chronic noncancer pain remains controversial, such use has increased substantially over the past few decades [1], as has prescribed availability of these medications [2]. The beneficial impact of this is presumably improved pain management for many patients. Unfortunately, one negative consequence of increased availability is that abuse of and addiction to prescription opioids has also increased dramatically over the past decade. A recent national survey finds that nearly 12 million persons (4.8%) 12 years of age or older indicate nonmedical use of prescription pain relievers in the past year [3]. The number of ER visits due to the nonmedical use of opioids has more than doubled from 2004 to 2008; from 144,600 to 305,900 visits, respectively [4]. The US death rate due to drug overdoses has never been higher and this increase is largely due to prescription opioid painkillers [5]. According to the annual national survey, 70% of nonmedically used analgesics are obtained from friends or family [3].

Most published statistics regarding nonmedical use/abuse of prescription opioids are limited to a general examination of any prescription opioid e.g., [3] or, at best, descriptions of one or two compounds such as oxycodone (usually OxyContin^® ^or other oxycodone preparation (e.g., Percocet^® ^or Percodan^®^) or the hydrocodone combination products (especially Vicodin^®^) (e.g., [6]). This likely reflects a primary interest in the most widely prescribed opioid compounds (namely oxycodone and hydrocodone) as well as the fact that some data streams do not differentiate among the various prescription opioid compounds (e.g., the Treatment Episode Dataset or TEDS: [7]). Similarly, discussions of ROAs employed by abusers of prescription opioids typically do not examine differential ROA patterns that may be characteristic of various products, compounds or formulations (e.g., [2,7,8]).

The premise of this article is that it is important to differentiate the relative risks of abuse of various prescription opioid compounds and formulations as well as the characteristic ROA patterns of the various compounds. The need for such specific data has increased due to two, relatively recent developments: the advent of the so-called abuse deterrent (ADF) or tamper resistant formulations (TRF) and the Food and Drug Administration's (FDA) recent efforts to employ Risk Evaluation and Mitigation Strategies (REMS) for specific prescription opioids and formulations.

Several pharmaceutical companies have begun to introduce ADFs or TRFs that are intended to decrease levels of abuse of prescription opioid medications (e.g., [9-13]). Many of these formulations propose some mechanism to thwart abusers' attempts to modify the tablet, capsule or patch in order to render the active ingredient immediately available for abuse and conducive for use via unintended or alternate ROAs (e.g., snorting/insufflation, injection) that have been associated with serious health consequences (e.g., [14-16]). Since these formulations are designed to resist tampering but can readily be abused by swallowing whole, the most accurate term to use is tamper resistant (TRF), which we use in this article. Note that at the time of this writing, no formulation has been granted a claim of either abuse deterrent or tamper resistant by the FDA. Clearly, evaluation of the public health impact of these TRFs is warranted once these products are on the market and available in communities to be abused. Given the long history of opioid abuse, it is not expected that the TRFs will eradicate abuse of prescription opioids or even tampering [11]. Thus, abuse deterrence or tamper resistance is generally discussed in terms of comparators; (i.e., abuse deterrent or tamper resistant compared to what? [17]). It will therefore be important to establish baseline relative risks of abuse of comparator compound(s) for a given TRF. And, since the focus of most TRFs is to inhibit unintended or alternate ROAs that require tampering, it is important to have established characteristic ROA patterns of comparator compounds or formulations in order to evaluate whether a TRF impacts the original ROA patterns of the comparator(s).

The second development suggesting the need to discriminate abuse patterns of compounds and formulations are recent efforts by the FDA to subject specific prescription opioids and formulations to REMS, as well as efforts to establish a classwide REMS for extended-release opioids [18]. Current REMS for prescription opioids, and the proposed classwide REMS, are applied to particular compounds and/or formulations (such as extended-release formulations). Thus, in principle, in order to measure the impact of these REMS, it is essential to differentiate abuse patterns of one compound or formulation from other compounds, since different compounds/formulations that may be subjected to a REMS have different a priori abuse patterns. Without such metrics it would be difficult to determine whether observed changes in abuse levels and ROA patterns due to REMS have occurred, and if so, whether the impact is on all drugs in a class or only for certain drugs. Furthermore, given the introduction of TRFs, there may be reason to go beyond the compound and general formulation (e.g., immediate-release [IR] versus extended-release[ER]) to ascertain differences in abuse patterns at the product specific level.

There are, to be sure, several articles that examine abuse patterns of specific compounds, formulations or products. For example, Kelly and colleagues (2008)[2] conducted a random telephone survey of households in the US. These authors differentiated 11 specific compounds and some formulations (i.e., combinations with acetaminophen) along with an "other" category. They reported the relative percentages of those who had taken one of these drugs in the past week. Their sample and methods did not address misuse or abuse, but rather served to report on the prevalence within the general population of individuals who had taken a prescription opioid for any reason (i.e., legitimate and illegitimate) in the past week. Another article by Rosenblum and colleagues (2007)[19] examined participants in 72 methadone maintenance treatment programs in 33 states. Respondents completed a checklist of lifetime and past 30 days abuse ("used to get high") of heroin and seven prescription opioids, including buprenorphine, fentanyl, hydrocodone, hydromorphone, oxycodone (ER and IR), methadone, morphine, and other opioid drug. They present the relative risks of abuse for respondents' primary problem and any abuse in the past 30 days for the compounds and formulations in their questionnaire. The presentation of ROAs in this study is confined to reports of injecting one's primary drug of abuse.

An extensive review of the public datasets administered by SAMHSA is beyond the scope of this brief review. However, two SAMHSA datasets do provide some compound and product-specific data: the Drug Abuse Warning Network (DAWN) dataset, which monitors drug-related visits to hospital emergency departments and drug-related deaths investigated by medical examiners and coroners, and the National Survey on Drug Use and Health [20], which provides national and state data on the extent and patterns of substance abuse (alcohol, tobacco, and illicit and prescription drugs) by conducting annual surveys of the general US population. One report from DAWN [21] examined relative rates of nonmedical use of six compounds (oxycodone, hydrocodone, methadone, fentanyl and hydromorphone) mentioned in emergency room visits, as well as change in number of mentions from 2004 to 2008. These datasets also collect information on ROAs, however, this is typically reported at the level of prescription opioids overall. We could find no report that distinguished ROA patterns among the various compounds or products.

The only published report, of which we are aware, that explicitly presents data on relative rankings of abuse of prescription opioid compounds and products, as well as compound-specific ROA patterns is Butler and colleagues' (2008)[22] report on the NAVIPPRO^® ^data stream, the ASI-MV^® ^Connect network. These authors present the relative percentages of individuals entering treatment for substance abuse at participating treatment centers across the country who report abuse specific compounds and products in the past 30 days. These data suggest that most prescription opioid abusers reported using a hydrocodone product in the past 30 days, followed closely by any oxycodone (both IR and ER), and followed more distantly by analgesic methadone, morphine, fentanyl and hydromorphone products. These authors report data showing that hydrocodone products are most always taken orally and almost never snorted or injected. Other compounds are also taken orally, but oxycodone ER products tend to be snorted and injected more often in this population of presumably hard core abusers, while morphine products are injected more often than taken orally. While these results are interesting and useful, there is no literature of which we are aware that specifically compares the relative risk of abuse of prescription opioid compounds and formulations. Nor is there a comprehensive comparison of ROA pattern differences among these compounds and formulations.

When considering the issue of relative abuse of compounds and formulations of prescription opioids, it is critical to consider how the raw counts of abuse cases or events are adjusted in order to compare risk of abuse across medications. In the literature on prescription opioid abuse, there is considerable discussion on this topic along with various proposals for the "best" denominator (e.g., [17,23,24]). We contend that abuse may be productively viewed from a variety of angles, since each adjustment may tell a different story regarding abuse patterns. Furthermore, the most "appropriate" adjustment likely depends on characteristics of the data source, and most importantly, the question or questions being asked of the data. Questions of prevalence usually address the likelihood that a given individual in some specified population will abuse the target substance (cf. [25]). Thus, one might examine the likelihood a product is to be abused in the general population or in a population of individuals known to abuse such drugs. Another important question relevant to prescription opioid abuse reflects the notion that the amount of abuse observed is strongly related to the prescribed availability within a community [26], raising questions of the level of abuse in a given community given the amount of prescribed drug in that community. Or, one might consider how likely it is that a prescription for a given analgesic will end up being abused. The answers to such questions often require data that are not readily available in the field of prescription opioid abuse, so that selection of suitable proxy measures (e.g., [17]) is required.

In the work reported here, we are interested in examining the unadjusted relative risks of abuse of seven prescription opioid compounds and, when appropriate, their immediate release and extended release formulations, similar to the relative rankings reported by Butler et al. (2008)[22]. We also go beyond these analyses to determine how these relative risks change when adjusted for the number of prescriptions written for the compared compounds/formulations. In a sense, this question asks: how likely is a particular prescription for an opioid analgesic to end up in the hands of an abuser? In addition, we provide descriptive information on patterns of abuse via routes of administration characteristic of the various prescription opioid compounds/formulations. We address these questions using data from a population of individuals entering substance abuse treatment programs who report abuse of these medications in the past 30 days.

## Methods

### Data sources

#### ASI-MV^® ^Connect

ASI-MV Connect is a proprietary data stream of the National Addiction Vigilance Intervention and Prevention Program (NAVIPPRO^®^) that collects data on substances used and abused by individuals in treatment for substance use disorders within a national network of substance abuse treatment centers. The Addiction Severity Index (ASI) is a standard intake assessment designed for use on admission to drug and alcohol treatment [27,28] that has demonstrated reliability and validity [29]. The Addiction Severity Index-Multimedia Version (ASI-MV^®^) is a computer-administered version of the ASI that has demonstrated good reliability (test-retest) along with discriminant validity for both English and Spanish versions [30-32]. The ASI-MV employs audio and video components to enhance respondent engagement in the assessment tasks and facilitates completion of the assessment by those with literacy issues. The ASI-MV Connect is a modified version of the ASI-MV in which respondents who indicate use/abuse of a prescription opioid are guided to questions about use and abuse of specific pharmaceutical products using screens with names (trade, generic, and slang names) and pictures of the products. Follow-up questions make specific inquiries for each product on all ROAs.

The patient-level de-identified data captured in the ASI-MV Connect are HIPAA (Health Insurance Portability and Accountability Act) compliant. Research conducted on these data are exempt from IRB policy [33].

Typically, the disadvantage of de-identified data, however, is that it prevents longitudinal analysis of cases. To address this issue, the ASI-MV Connect utilizes an algorithm which assigns each case a unique, 128-character identifier that is a concatenation of data entered by patients and are unlikely to change (e.g., gender, year of birth, mother's name, etc.). Using cryptographic techniques, the identifier is converted into a unique linking code for each patient and is maintained in the dataset but no longer reveals any elements of the personally identifying information. The nature of the ID permits linking of assessments by the same individual who completes the ASI-MV Connect assessment at different times and even at different places. Testing of a similar system with census data found an unduplicated rate of 99.845% [34]. The unique ID retains patient privacy while permitting longitudinal tracking of patients within and across treatment centers.

#### SDI Health LLC

SDI Health LLC provides data on prescriptions dispensed at the 3-digit ZIP code level on a monthly basis. SDI (Vector One National) is a national level projected prescription and patient-centric tracking service. Prescription data are obtained from a sample of approximately 59,000 pharmacies throughout the U.S. accounting for nearly all retail pharmacies, including national retail chains, mass merchandisers, pharmacy benefits managers and their data systems, and provider groups, and represent nearly half of retail prescriptions dispensed nationwide.

### Definition of abuse

Since prescription opioids are used legitimately with a prescription for pain, there is disagreement around what constitutes "abuse," per se, and how that is different from "misuse" of a prescription (e.g., [35]). In the case of individuals who are in substance abuse treatment, any strictly non-medical use of a mind altering substance is generally considered a relapse and would be classified as abuse. Thus, since some individuals in treatment for addictive disorders may also be prescribed and legitimately take medications, a series of questions establishes that the person has a current chronic pain problem and has taken prescribed opioid medication for pain in the past 30 days, that they have obtained their medications only from their own physician, and they have not used the drug via an alternate ROA. They are also asked if they have used a prescription opioid in the past 30 days "not in a way prescribed by your doctor, that is, for the way it makes you feel and not for pain relief." An algorithm based on answers to these questions identifies the patient as having engaged in non-medical use and are assumed to be abusing the medication.

### Medications selected for comparison

Although the ASI-MV Connect assessment differentiates medications at the product level, for present purposes specific products were aggregated to the compound and, within compound, to the respective immediate release (IR) and extended release (ER) versions of these compounds, as appropriate. Seven prescription opioid analgesic compounds and their IR and ER versions were selected for examination, resulting in a total of 11 different compound/formulations included in the analyses (Table 1). This list represents the primary Schedule II compounds prescribed in the US for pain, along with one Schedule III compound, hydrocodone, which is known to be widely prescribed and widely abused (e.g., [6,22]). Note that, during 2009, no ER hydromorphone was available in the US. Although methadone does not have an ER version, it is considered a long-acting opioid due to its long half-life (average half-life of twenty-four hours; [36]), and is therefore included with the extended release formulations. Extended release fentanyl products refer to the transdermal formulations.

**Table 1:** Compounds/formulations Included in the analyses

Generic Name	Immediate release	Extended release or long acting
**hydrocodone**	IR	NA

**oxycodone**	IR	ER

**fentanyl**	IR	ER/transdermal

**hydromorphone**	IR	Not available in US in 2009

**methadone**	NA	Long Acting

**morphine**	IR	ER

**oxymorphone**	IR	ER

### Statistical analyses

Data analysis was carried out in the following steps: (1) compute descriptive statistics of demographic variables to examine the sample characteristics; (2) fit two log-binomial regression models to estimate the unadjusted risk of abuse and prescription-adjusted risk of abuse of each IR and ER compound; and (3) fit a random effects binary logistic regression model to estimate the predicted probabilities of abusing each IR and ER compound by one of five ROAs, intended ROA (usually swallowing whole), inhalation or snorting, injection, chewing and then swallowing, and other. In addition to estimating the predicted probabilities from the random effects binary logistic regression model, we also estimated the predicted odds of abusing ER and IR morphine via each of ROA relative to the other compounds.

To carry out the second step, the original data set was structured such that each case line was associated with the proportion of sampled patients from one of the four US Census regions of the country (based on patient home 3-digit ZIP code) who endorsed abusing each compound. Since there were 11 compounds and 4 regions, the data set included exactly 11 × 4 or 44 cases. The first log-binomial model was fit to estimate the unadjusted risk of abuse of each compound, with the categorical indicator variable (compound) as the independent variable and the number of abuse cases per compound per region over the total number of cases sampled per compound per region as the dependent variable. The second log-binomial model was fit to estimate the prescription-adjusted risk of abuse of each compound, with the categorical indicator variable (compound) as the independent variable, log (number of prescriptions dispensed per region/100,000) as the offset, and the number of abuse cases per compound per region over the total number of cases sampled per compound per region as the dependent variable. A log-binomial model was selected over the more standard Poisson model to estimate risk of abuse since there was a finite number of patients sampled, which varied substantially across regions. The log-binomial model can directly account for the varying finite number of cases sampled in the dependent variable (38 events/total # of trials), while still accounting for an offset variable. Of note, in this paper we refer to the unadjusted estimates derived from the first log-binomial model as "risk" estimates, since these estimates reflect the number of abuse cases over the number of cases sampled. To be consistent, we also describe the prescription-adjusted estimates derived from the second log-binomial model as "risk per 100,000 prescriptions" estimates.

To carry out the 3rd step, the data set was structured such that each case line was associated with a patient's yes = 1/no = 0 response on abuse of a compound through a specific ROA. A random effects binary logistic regression model was fit with the categorical indicator variables (compound, route, and compound-BY-route) as the independent variables and the binary variable (yes/no abuse via a specific ROA) as the dependent variable. A random intercept was incorporated in this model to account for co-variation due to multiple observations per patient, since each patient is measured on abuse via each route of administration for each compound. This model was fit using only data from substance abuse treatment patients who reported having abused the compound(s). Limiting the sample in this way allowed us to estimate the probability of abusing a particular compound via a specific route of administration among those who reported having abused that particular compound. Analyses were performed using the generalized linear modeling procedure (GENMOD) and the generalized linear mixed modeling (GLIMMIX) procedure in SAS/STAT 9.22 software.

## Results

### Respondent characteristics

**Figure 1:**
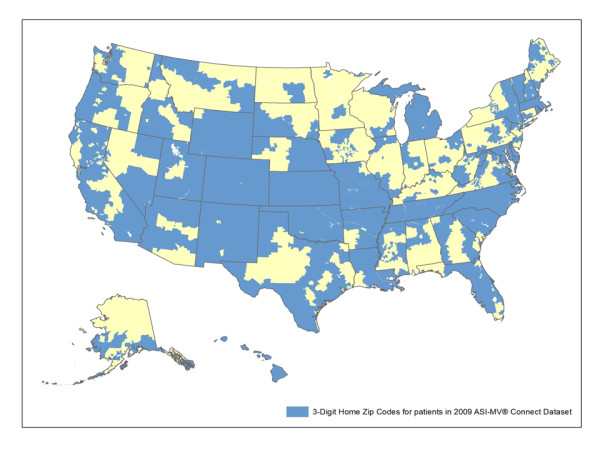
**Map of Home 3-digit ZIP Codes of 2009 ASI-MV Connect Patients**. Shaded blue regions show 3-digit home zip codes for patients included in the 2009 ASI-MV Connect database.

Data from 69,002 patients in substance abuse treatment within the ASI-MV Connect system were collected during the calendar year of 2009. Of the total sample, 13.3% represented follow-up assessments and were not included in the analyses, leaving a total N of 59,792 unique patients included in the analyses. Of these, 14.6% reported abusing at least one prescription opioid in the past 30 days and 4.8% reported appropriate medical use of a prescription opioid in the past 30 days. With respect to geographic coverage, data are collected on patients' 3-digit home ZIP code. In the total sample, patients reside in 571 unique 3-digit ZIP codes (64% of 886 U.S.3-digit ZIP codes), while individuals reporting past 30 day abuse of any prescription opioid reside in 354 unique 3-digit ZIP codes (38%; see Figure 1). Table 2 presents respondent characteristics separately for the entire sample of unique patients and those who report abusing prescription opioids in the past 30 days. As can be seen, the prescription opioid abuser sample contains a greater percentage of women and whites and fewer African Americans than the ASI-MV Connect substance abuse treatment sample as a whole.

**Table 2:** Demographic Characteristics of Participants

	Entire Sample N = 59,792		Prescription Opioid Abusers N = 8,739	
**Characteristic**	**M**	**SD**	**M**	**SD**

Age	33.7	11.5	33.0	10.8

	**N**	**%**	**N**	**%**

Gender				

Male	40,147	67.1	5,178	59.3

Female	19,644	32.9	3,561	40.7

Race				

Caucasian	31,690	53.0	5,755	65.9

Hispanic/Latino	11,212	18.8	1,534	17.6

African American	13,063	21.8	1,092	12.5

Native American/Alaskan Native	3,407	5.7	301	3.4

Asian/Pacific Islander	415	0.7	55	.6

Current treatment episode prompted by criminal justice system	36,984	62.0%	3,471	39.9

### The ASI-MV Connect Network

**Figure 2:**
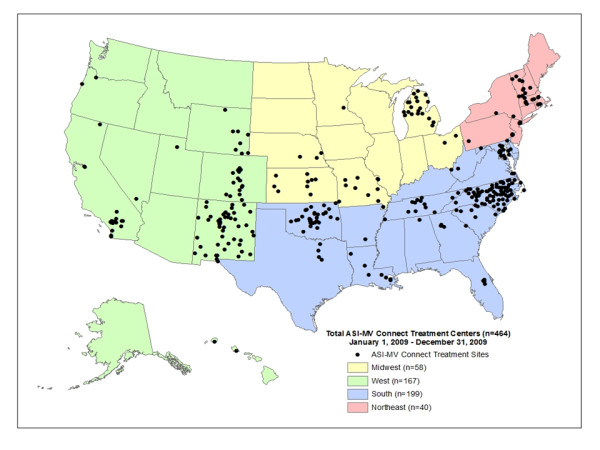
**Map of the ASI-MV Connect Network of Participating Treatment Facilities**.

Treatment sites purchase the ASI-MV Connect software, which generates a psychosocial report and other documentation that is important clinically. As such, this assessment is part of the clinical flow and is not a separate survey or questionnaire (Butler et al., 2008). All 59,792 unique patients assessed during 2009 at 464 ASI-MV Connect network treatment facilities in 34 states were included in the total sample. This can be compared with, for example, 2009 data from the SAMHSA National Survey of Substance Abuse Treatment Services (N-SSATS; [37], the annual census of substance abuse treatment facilities in the US, which reported a one-day census of 1,182,077 clients enrolled in substance abuse treatment in 13,513 facilities nationwide. Figure 2 presents a map of the geographic distribution of the treatment facilities within the ASI-MV Connect network across the US. These treatment facilities are a combination of state, federal and local (e.g., county) government agencies as well as and private non-profit and private for-profit organizations. During 2009, payors for about 20% of the patients were public sources, with about 4% commercial payors, 43% "self-pay", 9% uninsured or exhausted benefits, and 24% other. About 16% of patients were in residential or inpatient settings, 34% in outpatient/non-methadone, 2% in methadone treatment programs, 34% in a corrections setting (e.g., drug court, probation/parole and DUI/DWI evaluation) and 14% other.

### General Abuse

**Figure 3:**
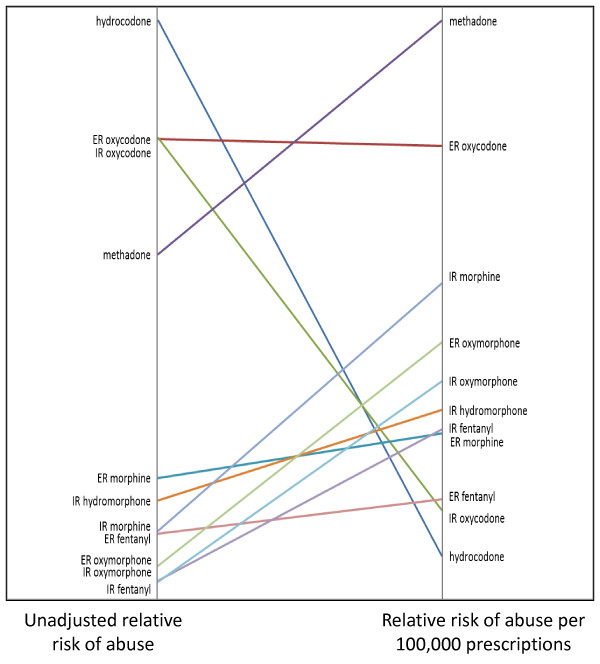
**Ladder Graph of Normalized Unadjusted and Adjusted Abuse Risk Estimates for 11 Prescription Opioid Compounds and Formulations**. This figure presents a ladder graph that normalizes the unadjusted and adjusted risk estimates in Table 3 to a range of 0 and 1. This graph illustrates how the estimates change for each compound/formulation when adjusting for prescription volume.

Results from the first log-binomial model revealed that the highest unadjusted risk of abuse was associated with (1) hydrocodone, followed by (2) IR oxycodone, (3) ER oxycodone, (4) methadone, (5) ER morphine, (6) IR hydromorphone, (7) IR morphine, (8) ER fentanyl, (9) ER oxymorphone, (10) IR fentanyl, and (11) IR oxymorphone (Table 3). After adjusting for prescriptions in the second log-binomial model, (1) methadone was estimated to be the most highly abused compound, followed by (2) ER oxycodone, (3) IR morphine, (4) ER oxymorphone, (5) IR oxymorphone, (6) IR hydromorphone, (7) IR fentanyl, (8) ER morphine, (9) ER fentanyl, (10) IR oxycodone and (11) hydrocodone (Table 3). It is clear that when one adjusts for prescriptions, several compounds that are initially estimated to have comparatively low abuse (e.g., IR morphine) are estimated to have much higher relative levels of abuse. Moreover, based on the second log-binomial model, most of these prescription-adjusted abuse risk estimates are significantly different from each other (Table 4). Figure 3 presents a ladder graph that normalizes the unadjusted and adjusted risk estimates in Table 3 to a range of 0 and 1. This graph illustrates how the estimates change for each compound/formulation when adjusting for prescription volume.

**Table 3:** Unadjusted Abuse Risk, Abuse Risk per 100,000 Prescriptions, and Total Number of Prescriptions per 100,000

Compound	Abuse Risk	(+) Abuse Risk **per 100,000 Prescriptions**^£^	Total Number of Prescriptions per 100,000
hydrocodone	0.473	0.0022	585.620

IR oxycodone	0.375	0.0055	211.821

IR fentanyl	0.005	0.0114	1.212

IR hydromorphone	0.072	0.0129	18.433

IR morphine	0.047	0.0220	6.675

IR oxymorphone	0.003	0.0150	0.706

ER oxycodone	0.374	0.0320	37.167

ER fentanyl	0.044	0.0063	22.934

Methadone	0.278	0.0411	20.028

ER morphine	0.091	0.0111	26.059

ER oxymorphone	0.017	0.0177	2.896

^£^To show the differences in prescription-adjusted risks, it was necessary to round to the 4th decimal place due to the magnitude of the prescription volume for some compounds.

**Table 4:** Prescription-Adjusted^£ ^Relative Risk of Abusing each Compound

Compound	hydrocodone	IR oxycodone	IR fentanyl	IR hydromorphone	IR morphine	IR oxymorphone	ER oxycodone	ER fentanyl	methadone	ER morphine	ER oxymorphone
**hydrocodone**	1.000	--	--	--	--	--	--	--	--	--	--

**IR oxycodone**	2.494^¥^	1.000	--	--	--	--	--	--	--	--	--

**IR fentanyl**	5.154^¥^	2.066^¥^	1.000	--	--	--	--	--	--	--	--

**IR hydromorphone**	5.828^¥^	2.336^¥^	1.131	1.000	--	--	--	--	--	--	--

**IR morphine**	9.976^¥^	3.999^¥^	1.936^¥^	1.712^¥^	1.000	--	--	--	--	--	--

**IR oxymorphone**	6.781^¥^	2.718^¥^	1.316	1.164	0.680^τ^	1.000	--	--	--	--	--

**ER oxycodone**	14.520^¥^	5.821^¥^	2.817^¥^	2.492^¥^	1.456^¥^	2.141^¥^	1.000	--	--	--	--

**ER fentanyl**	2.846^¥^	1.411^τ^	0.552^£^	0.488^¥^	0.285^¥^	0.420^¥^	0.196^¥^	1.000	--	--	--

**methadone**	18.645^¥^	7.475^¥^	3.617^¥^	3.199^¥^	1.869^¥^	2.750^¥^	1.284^¥^	6.551^¥^	1.000	--	--

**ER morphine**	5.051^¥^	2.025^¥^	0.980	0.876^τ^	0.506^¥^	0.745	0.348^¥^	1.775^¥^	0.271^¥^	1.000	--

**ER oxymorphone**	8.010^¥^	3.211^¥^	1.554^τ^	1.374^£^	0.803^τ^	1.181	0.552^¥^	2.814^¥^	0.430^¥^	1.586^¥^	1.000

^£ ^per 100,000 Prescriptions

^¥^p < .0001

^£^p < .001

^τ^p < .05

**Figure 4:**
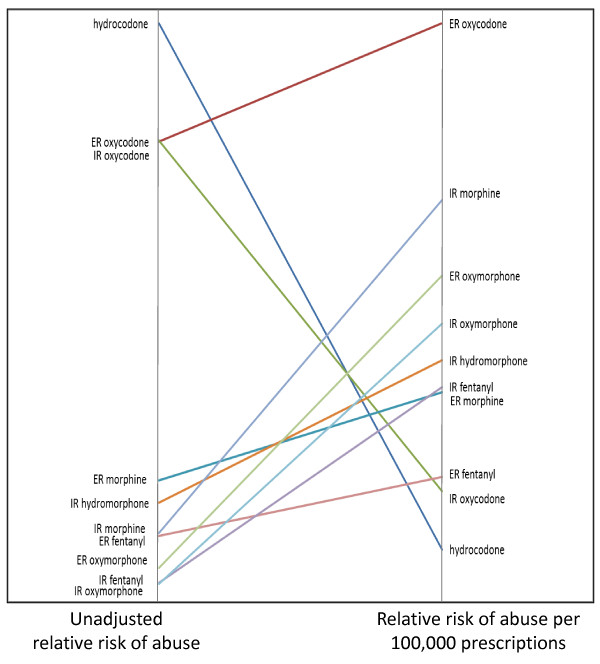
**Ladder Graph of Normalized Unadjusted and Adjusted Abuse Risk Estimates for the Prescription Opioid Compounds and Formulations Excluding Methadone**. This figure presents the same data as Figure 3 albeit without methadone in order to present clearly the impact of removing methadone from the analyses.

The increase in the relative abuse risk of methadone was somewhat unexpected and, upon reflection, may be related to some of the challenges presented by unique characteristics of methadone, particularly in the context of a substance abuse treatment population. Like the other prescription opioid compounds examined here, methadone is used for the treatment of pain, however, it is also used medically as part of methadone maintenance programs to help those with opioid addiction function more effectively. Methadone dispensing in opioid treatment programs (OTPs) and other formulations of methadone (i.e., elixir) may have affected the above analyses in unknown ways. However, methadone is a long acting opioid and as such is also attractive for abuse by these populations. Figure 4 presents the same the data as Figure 3 albeit without methadone in order to present clearly the impact of removing methadone from the analyses.

### Abuse via Specific ROAs

Results from the random effects binary logistic regression model revealed varying patterns of abuse across compounds (See Table 5 for the model-predicted probabilities of abusing each compound through each ROA as well as the actual number of abuse cases associated with each compound through each ROA). As seen in Table 5, while on one hand hydrocodone is most likely to be abused through the intended/swallowed whole route (prob. = 0.896), morphine (prob. IR = 0.558, prob. ER = 0.451) and IR hydromorphone (prob. = 0.554) have a comparatively high probability of being abused by injection.

**Table 5:** Frequency (n) and Predicted Probability with 95% CI of Specific Routes of Administration by Compound/Formulation

	Immediate release			Extended-release/long-acting^1^		
Generic name	Number	Predicted Probability	95% CI	Number	Predicted Probability	95% CI
**Hydrocodone**	4,136	-	-	-	-	-
Intended ROA	3,668	0.896	0.887,0.905	-	-	-
Injection	49	0.010	0.007,0.013	-	-	-
Inhalation	795	0.171	0.160,0.183	-	-	-
Chew	759	0.163	0.152,0.175	-	-	-
Other	240	0.048	0.042,0.055	-	-	-
**Oxycodone**	3,279	-	-	3,271	--	--
Intended ROA	2,671	0.824	0.810,0.837	2,034	0.621	0.603,0.639
Injection	208	0.052	0.045,0.059	803	0.221	0.207,0.236
Inhalation	932	0.260	0.245,0.276	1,502	0.444	0.426,0.463
Chew	650	0.175	0.162,0.188	605	0.162	0.150,0.175
Other	242	0.063	0.056,0.072	346	0.089	0.080,0.099
**Fentanyl**	39	-	-	383	-	-
Intended ROA	5	0.081	0.032,0.090	33	0.060	0.043,0.085
Injection	4	0.062	0.022,0.163	85	0.171	0.138,0.210
Inhalation	7	0.120	0.054,0.244	6	0.010	0.004,0.023
Chew	7	0.119	0.054,0.243	39	0.072	0.052,0.098
Other	27	0.630	0.453,0.778	270	0.676	0.623,0.725
**Hydromorphone**	626	-	-	-	-	-
Intended ROA	208	0.295	0.259,0.333	-	-	-
Injection	361	0.554	0.512,0.595	-	-	-
Inhalation	153	0.208	0.178,0.242	-	-	-
Chew	49	0.061	0.046,0.080	-	-	-
Other	51	0.064	0.048,0.084	-	-	-
**Methadone**	-	-	-	2,426	-	-
Intended ROA	-	-	-	2,009	0.836	0.820,0.850
Injection	-	-	-	179	0.060	0.052,0.070
Inhalation	-	-	-	366	0.129	0.116,0.143
Chew	-	-	-	351	0.123	0.111,0.137
Other	-	-	-	350	0.123	0.111,0.136
**Morphine**	408	-	--	792	-	-
Intended ROA	146	0.332	0.286,0.381	336	0.389	0.354,0.425
Injection	232	0.558	0.506,0.608	382	0.451	0.415,0.488
Inhalation	81	0.173	0.140,0.213	222	0.242	0.213,0.274
Chew	45	0.092	0.069,0.123	89	0.089	0.072,0.109
Other	33	0.066	0.047,0.093	38	0.036	0.026,0.050
**Oxymorphone**	30	-	--	149	-	-
Intended ROA	14	0.388	0.226,0.578	47	0.249	0.187,0.324
Injection	6	0.129	0.054,0.280	12	0.055	0.031,0.096
Inhalation	18	0.535	0.344,0.716	114	0.738	0.654,0.807
Chew	4	0.081	0.028,0.212	23	0.109	0.072,0.163
Other	4	0.089	0.031,0.224	4	0.120	0.008,0.049

^1^Intended routes of administration: Fentanyl ER, patch; Fentanyl IR, sublingual; all others, swallowed whole. Methadone is the only long-acting opioid; all other opioids are extended-release.

It is certainly possible when fitting the random effects binary logistic regression model in the GLIMMIX procedure to estimate the odds of abusing one compound via a specific route relative to another compound. As an example, Tables 6 and 7 provide the model-predicted odds of abusing IR and ER morphine through each ROA relative to all other compounds. Examining these tables, it becomes clear that the ROAs associated with IR and ER morphine can be significantly differentiated from other drugs. In particular, morphine in either IR or ER formulation is more likely to be abused via injection than all other compounds/formulation with the exception of hydromorphone.

**Table 6:** Odds of Abusing IR Morphine Via Specific Routes of Administration Relative to other Compounds

	Route of Administration				
**IR morphine *vs*.**	**Intended**	**Snort**	**Inject**	**Chew**	**Other**

Hydrocodone	0.058^¥^	1.014	125.00^¥^	0.522^¥^	1.404

IR oxycodone	0.106^¥^	0.600^¥^	23.256^¥^	0.480^¥^	1.050

IR fentanyl	5.682^£^	1.537	19.231^¥^	0.750	0.042^τ^

IR hydromorphone	1.188	0.797	1.014	1.570^τ^	1.043

IR oxymorphone	0.785	0.182^¥^	8.479^¥^	1.161	0.732

ER oxycodone	0.303^¥^	0.262^¥^	4.443^¥^	0.526^τ^	0.731

ER fentanyl	7.717^¥^	20.806^¥^	6.111^¥^	1.312	0.034^¥^

Methadone	0.098^¥^	1.416^τ^	19.590^¥^	0.723	0.508^£^

ER morphine	0.780	0.656^τ^	1.533^£^	1.041	1.887^τ^

ER oxymorphone	1.498	0.075^¥^	21.552^¥^	0.829	3.561^τ^

^¥^p ≤ .0001

^£^p ≤ .001

^τ^p ≤ .05

**Table 7:** Odds of Abusing ER Morphine via Specific Routes of Administration Relative to other Compounds

	Route of Administration				
**ER morphine *vs*.**	**Intended**	**Snort**	**Inject**	**Chew**	**Other**

hydrocodone	0.074^¥^	1.546^¥^	83.333^¥^	0.502^¥^	0.744

IR oxycodone	0.136^¥^	0.908	14.925^¥^	0.461^¥^	0.556^τ^

IR fentanyl	7.246^¥^	2.342	12.500^¥^	0.720	0.022^¥^

IR hydromorphone	1.522^£^	1.215	0.662^£^	0.663^τ^	0.552 ^τ^

IR oxymorphone	1.006	0.278^τ^	5.525^£^	1.115	0.388

ER oxycodone	0.389^¥^	0.399^¥^	2.899^¥^	0.506^¥^	0.387^¥^

ER fentanyl	9.901^¥^	31.250^¥^	3.984^¥^	1.261	0.018^¥^

methadone	0.125^¥^	2.160^¥^	12.821^¥^	0.694^τ^	0.269^¥^

ER oxymorphone	0.521^τ^	0.114^¥^	14.060^¥^	0.797	1.886

^¥^p ≤ .0001

^£^p ≤ .001

^τ^p ≤ .05

## Discussion

This paper presents the relative abuse risks of 11 prescription opioid compounds/formulations, both unadjusted as well as adjusted by the number of retail pharmacy-dispensed prescriptions for a particular high risk sample of substance abusers in treatment. Compound/formulation patterns of abuse via specific ROAs were also examined. Self-report data were drawn from nearly 60,000 substance abuse treatment patients who completed the ASI-MV Connect assessment at one of the 464 substance abuse treatment centers in the ASI-MV Connect network. In the present study, the unadjusted risks observed replicated the general findings of other studies. For example, Rosenblum and colleagues (2007)[19] in their survey of prescription opioid and heroin abusers in methadone maintenance programs found that both groups reported highest abuse (ever and in past 30 days) of hydrocodone as well as ER and IR oxycodone at similar levels. These three were followed by methadone, morphine, hydromorphone and fentanyl. Although these authors did not distinguish ER from IR morphine, their relative ranking of the drugs maps well with the order found in this study (see Figure 3). The DAWN report [21] found a similar pattern of the six compounds on which they reported, such that oxycodone products were highest followed closely by hydrocodone, then methadone and morphine, with fentanyl having somewhat larger numbers than hydromorphone. The relative rankings of compounds and formulations observed here are also similar to those reported by Butler and colleagues (2008)[22] who used ASI-MV Connect data collected between November 2005 and July 2008. Since the data used in this study are from 2009 only, it seems likely that the observed relative rankings are stable over time. Hydrocodone products were reported as most abused in the past 30 days, followed by ER and IR oxycodone products, methadone and ER morphine products, hydromorphone, IR morphine, ER fentanyl and IR fentanyl products. Finally, Kelly and colleagues (2008)[2] looked at a very different population, namely a general public sample, and they used a household-based, telephone survey asking about any use (including legitimate use). These authors reported hydrocodone products to be more widely used than oxycodone products, again followed by methadone, with fentanyl and morphine at the same, lower level. Taken together, these results compare favorably with the present findings of relative risk of abuse based on unadjusted values observed in the present study. As these studies involve different populations, timeframes, and data collection methods, the general correspondence of findings suggest a certain robustness of the relative degree to which various prescription opioid compounds/formulations are used or abused in the US.

One goal of the present study was to go beyond the prior work to examine the effect on relative risks of abuse of prescription opioid compounds and formulations by adjusting for the number of prescriptions written in the local areas where the abusers reside. This question was stimulated in part by awareness that risk of abuse appears to be related to the prescribed availability within a community (e.g., [26]). Another major reason for investigating adjusted risks of abuse is the magnitude of differences between prescriptions for the different compounds and formulations. As can be seen in Table 3, the compound/formulation with the least amount of prescriptions in the patient home ZIP codes represented here (IR oxymorphone) has about 825 times fewer prescriptions than hydrocodone. This, in turn, raised the question of how relative risks of abuse of the prescription opioid compounds/formulations would change if level of prescribed availability were taken into account. It is not surprising that abuse risks are associated with prescription volume, since a drug must first be available before it can be abused. However, in practical terms, it may be helpful to examine the impact of prescription volume on abuse for particular compounds/formulations. From the prescriber's perspective, such an analysis may capture the extent to which a given prescription is likely to end up in the hands of an abuser. Consistent with this reasoning, the present study revealed clear differences in the impact of prescription volume on risk of abuse of the various prescription opioid compounds/formulations observed in the ASI-MV Connect data. As seen in Figure 3, the impact of prescription volume on abuse risks is largest for two of the most widely prescribed and widely abused compounds/formulations, hydrocodone and IR oxycodone. These drugs decline from the top of the ranking to the bottom after adjusting for prescription availability. This suggests that, despite the well-known high levels of abuse of these drugs, on a prescription-by-prescription basis, they are not as likely to be abused. Shifts in the other direction are seen for methadone and IR morphine, implying that the converse may be true for these drugs--namely prescriptions for these drugs may be more likely to end up being abused. Methadone, in this analysis, increased dramatically in abuse risk as did ER oxycodone. The risk of abuse of ER morphine increases slightly when adjusted for prescription volume. When considered together with IR morphine, this suggests a somewhat greater likelihood of abuse of any morphine product on a prescription-by-prescription basis. ER morphine, however, falls in the overall ranking from an unadjusted position of fifth drug abused to eighth in the analyses adjusted for prescription volume, behind several other, much less often prescribed compound/formulations (e.g., ER oxymorphone, IR oxymorphone, IR hydromorphone, and IR fentanyl). ER fentanyl (transdermal fentanyl), like ER morphine increases somewhat in absolute terms but is only above IR oxycodone and hydrocodone in the ranking of adjusted risk of abuse.

The finding of differential impact of prescribed volume on different prescription opioid compounds and formulations may have a variety of explanations. The large decline in the relative ranking of adjusted abuse risks for hydrocodone and IR oxycodone may be something of an artifact of the fact that these drugs are very widely prescribed and much more so than any of the other compound/formulations included in this study. Commonly prescribed for acute pain and minor surgery, these medications are likely to be found in many households in the US. When adjusting levels of prescription opioid abuse by prescription volume values with such large differences between the drugs compared, dramatic shifts in the adjusted levels may be expected. The low adjusted abuse risks of hydrocodone and IR oxycodone do not suggest that these drugs present less public health concern. Rather, we would conclude that, on a prescription-by-prescription basis, these drugs are comparatively less likely to be abused. In contrast to hydrocodone and IR oxycodone, many of the other opioid analgesics examined here are intended for and presumably prescribed for much smaller populations, such as chronic pain patients, and for specialized purposes such as breakthrough pain in highly opioid tolerant pain patients (e.g., IR fentanyl products). The adjusted risks for abuse suggest that these more difficult to obtain products (based on lower prescribed volume) are more abused in the ASI-MV Connect population than would be expected based on availability alone. This may suggest that these products are highly sought after and successfully obtained by the hard-core abusers represented in this treatment population. These data also suggest that a given prescription for one of these prescription opioids that are presumably highly desirable for abuse may be more likely to end up involved in abuse activity.

As noted in the Results section, methadone presents some unique challenges when compared directly with other prescription opioids. Current ASI-MV Connect screens for methadone present pictures and names of methadone preparations that come in pill or "wafer" forms. Almost half (44.5%) of the methadone abuse cases in this ASI-MV Connect sample indicated abuse of methadone by selecting only the "other not shown" category. Examination of 2010 data, where the ROA option of "drinking" is available, suggests that this option is chosen by the preponderance of respondents who select the other methadone option. This, in turn, suggests that these respondents may be using the solution or elixir formulation of methadone. Another issue regards the extent to which retail pharmacy volume as captured by the SDI Health data accurately depicts "prescription volume" for methadone in a way that is comparable to the other compounds and formulations examined here. Finally, given the use of methadone as a treatment modality in substance abuse treatment, it is difficult to know the extent to which respondents misidentified such use in the past 30 days as misuse. Examination of the data suggests that at least a quarter of respondents who indicated use of methadone as "other not shown" also indicated use by an alternate ROA, such as snorting or injecting. However, it is possible that those who are indicating they "swallowed" methadone are doing so as part of their treatment. The present configuration of ASI-MV Connect questions do not allow for a clear differentiation of individuals using methadone as part of their treatment. Changes in the screens are planned to allow for this differentiation in the future. For present purposes, however, the findings reported here regarding the impact of prescription volume on relative abuse risk estimates for methadone should be interpreted cautiously. These issues may be important considerations when evaluating the suitability of methadone as a candidate comparator for TRFs of other prescription opioid compounds. As illustrated in Figure 4, the relative standing of the prescription opioids presented without methadone reveals ER oxycodone as the compound/formulation with the greatest risk level after adjusting for prescription volume. In this Figure, the other compounds/formulations retain their relative positions with respect to ER oxycodone.

We also intended to describe different route of administration (ROA) patterns of the prescription opioids examined in this study. The findings here are consistent with those reported by Butler and colleagues (2008)[22] who presented ROA patters for hydrocodone, oxycodone, morphine, methadone and fentanyl. These authors found hydrocodone to be mostly abused orally, oxycodone mostly abused nasally (by snorting or inhalation), morphine mostly likely injected, and fentanyl to be most likely smoked or "other." These findings are similar to the ones presented here, although the present analyses examine more compounds/formulations. In the present study, "oral ingestion" was more precisely broken down into swallowing whole (the "intended" route for all drugs except the fentanyl products) and chewing. The ASI-MV Connect now collects data on dissolving in mouth like a cough drop and drinking after dissolving in liquid, although these options were added in 2009 and not available for entire year examined here.

In the present study, it was clear that hydrocodone, IR oxycodone, and methadone had high levels of respondents (> .80 predicted probability) reporting swallowing the drug whole (intended ROA). Oxymorphone IR and ER had the highest levels of abusers reporting inhalation (prob. = .54 and prob. = .74 respectively) with abusers of ER oxycodone having a predicted probability of inhalation of about .44. As Butler et al. (2008)[22] observed, morphine abusers tend to inject the drug, with IR morphine having a .56 predicted probability of injection and ER morphine at .45. Examination of the odd ratios comparing morphine (IR and ER) ROAs with all other drugs, highlights that morphine is significantly more likely to be injected than any other prescription opioid, with the exception of IR hydromorphone which had a predicted probability of injection of .55 (see "inject" column in Tables 6 and 7). Of note also is that IR morphine was significantly more likely to be injected than ER morphine. It is possible, given the consistency with patterns observed in earlier analyses [22] that the ROA patterns observed in this study are robust over time and reliably differentiate certain compounds/formulations. Such baseline information will be essential when evaluating TRFs of prescription opioid compounds. As noted above, TRFs are intended to inhibit efforts to modify the product to make its active ingredients available for alternative ROAs, such as snorting or injection. The extent to which a TRF can be determined to be successful will require a clear understanding of the ROA patterns characteristic of the TRF's parent drug or other comparators. Clearly, a TRF whose parent product is rarely injected will be unlikely to have a large impact on its use by that ROA. The present analyses are a step in the direction of delineating such ROA patterns for specific compounds and their ER/IR formulations.

There are several limitations of the present study. To begin with, important limitations of the ASI-MV Connect data should be highlighted. These data represent self-reports of persons entering treatment for substance use disorders. Self-report data are subject to recall bias or reluctance to report accurately. Despite this, it is unclear what other source of information about use and routes of administration can be reliably obtained. Over the years, research continues to support the reliability and validity of self-report of patients entering treatment (e.g., [38-43]). Although such literature generally supports the validity of self-report, it should be acknowledged that a few studies have found self-reported use to under-report drug use (e.g., [44,45]). A further consideration is that individuals in this particular patient population have an acknowledged difficulty with substance abuse--a difficulty that has developed to the degree of necessitating treatment--and thus they may have less motivation to lie about their drug abuse in comparison with people who are not in treatment. In addition to the general support for the validity of self-reported substance use in the treatment setting, there is evidence that reporting via computer self-administration is as valid as reporting to a live interviewer. Where discrepancies exist, computer self-administration tends to elicit reports of more, rather than fewer, psychosocial and substance use problems [46]. Finally, the ASI-MV Connect assessment uses a methodology for questioning respondents about use/abuse of particular prescription medications that is similar to methods employed by the NSDUH survey [3]. NSDUH utilizes pictures of prescription products, names, slang and so forth as well as other widely accepted methodological practices for increasing the accuracy of self-reports, such as audio computer-assisted self-interviewing (as does the ASI-MV Connect). Examinations of these NSDUH methods have shown that they reduce reporting bias [47] in general populations.

Another limitation of the ASI-MV Connect data is that this dataset does not draw from a probability-based sample and, while having broad, national reach, does not provide comprehensive coverage of the US. The data collected by the ASI-MV Connect system are intended to provide sentinel population surveillance of substance abuse patterns in the US, but these data are yet to achieve national representativeness. The presented results are not nationally representative and are not intended to be used for estimating national incidence and prevalence rates. Furthermore, the population represented is not randomly selected. It consists of those who seek treatment for substance abuse and who have access to a substance abuse facility. The sample utilized is a convenience sample of patients assessed at treatment facilities that are part of the ASI-MV Connect network. The sample does not represent individuals who misuse or abuse prescription opioids but are not in treatment, nor does it include those in treatment but at treatment facilities not included in the ASI-MV Connect network, and the findings may not be generalizable to all patients with substance use disorders in treatment. Approximately 60% of cases in the ASI-MV Connect data (about 40% of the prescription opioid abusers--see Table 2) represent individuals whose treatment episode has been prompted by the criminal justice system. Thus, this database may have a socioeconomic bias against those who do not have access to such care.

These aspects of the ASI-MV Connect data serve as unavoidable limitations to any effort to establish population-based estimates. We believe, however, the present effort to examine relative risks of abuse and to describe abuse patterns observed in a saturated population, the ASI-MV Connect data may allow reliable estimates of large trends in abuse that would be relevant to the evaluation of TRFs and REMS. This is supported by the consistency with which the relative risks of abuse reported here and those reported in the other studies using different methods and populations, as mentioned above. Furthermore, the ASI-MV Connect dataset is the only source of data that provides systematic, prospective, and comprehensive information at the product-specific level necessary to answer questions regarding route of administration and other abuse patterns. Such information will be essential in addressing specific questions around tamper resistance and the effectiveness of REMS. Nevertheless, limitations of the data are acknowledged and present results should not be generalized beyond the population sampled. With this in mind, it should be noted that similar limitations apply to all public health data streams. Mortality data, for instance, suffer from underreporting and a lack of standardized procedures for attributing and coding poisoning deaths [48-50], yet these data have been used to support nationwide alerts from the FDA [51,52].

On a final note, the evaluation of tamper resistance and the effectiveness of REMS will require analysis of a variety of available data streams. It is unlikely that any single data stream alone will capture all relevant data to necessary to adequately evaluate misuse and abuse of prescription opioids [24]. Other methods, such as laboratory testing of abuse liability, could be particularly useful in evaluating tamper resistant properties of new formulations [53]. However, the FDA has made clear that any product claims of abuse deterrence or tamper resistance would not be made without "long-term epidemiological data from community-based observational studies that document changes in abuse and addiction and the consequences of those behaviors" [54]. Such epidemiological data will necessarily require samples of saturated populations such as those in substance abuse treatment and will need to obtain product-specific and route-specific data.

Finally, it is worth noting that while log-binomial models are recommended to estimate risk, these models are prone to either non-convergence or converging to invalid estimates (e.g., predicted probabilities greater than one) [55]. As generally recommended, we monitored model convergence and confirmed that all predicted probabilities fell within the bounds of 0 and 1. Also, use of maximum likelihood estimation to fit logistic regression models tends to produce unreliable estimates when the number of events (or nonevents) is small for some categories (e.g. injection of IR fentanyl). As a result, very low predicted probabilities estimated from the random effects logistic regression model should be interpreted with caution. While exact logistic regression has been proposed for such scenarios, this approach was deemed infeasible and inappropriate since (1) several of the other categories were associated with a reasonably large number of events (e.g. injection of IR morphine) and (2) co-variation among observations due to repeated measures was present.

## Summary and Conclusions

This study provides a comprehensive examination of the relative risks of abuse and ROA patterns of 11 compounds and formulations of prescription opioids in an at risk population of substance abusers in treatment. Using data from the ASI-MV Connect network of treatment centers across the country, relative risks of abuse were examined using unadjusted risks (based on the number of abusers of a particular compound/formulation relative to other prescription opioid abusers) and after adjusting for prescription volume. Results suggest that some drugs known to be widely abused, especially hydrocodone and IR oxycodone products, are abused less often than their prescribed volume would predict, while other drugs, such as methadone, morphine, hydromorphone, fentanyl and oxymorphone, are abused more often than their prescribed volume would predict. In addition, these data were examined to elicit ROA patterns that distinguish the various compounds/formulations, some of which (e.g., hydrocodone, IR oxycodone, methadone) tend to be used through the intended ROA (i.e., swallowed whole), while others (morphine, hydromorphone) are significantly more likely to be injected than other prescription analgesics. Establishing baselines of abuse risk and ROA patterns is necessary in order to adequately test the impact of the recently approved and marketed TRFs and to evaluate the effectiveness of classwide REMS for prescription opioids.

## Competing interests

The authors declare that they have no competing interests.

## Authors' contributions

SFB, RB, TAC, and TD participated in the design of the study and performed the statistical analysis. SFB, RB, and SHB conceived the study, and participated in its design and coordination and helped to draft the manuscript. All authors read and approved the final manuscript.
